# dFOXO-independent effects of reduced insulin-like signaling in *Drosophila*

**DOI:** 10.1111/j.1474-9726.2011.00707.x

**Published:** 2011-10

**Authors:** Cathy Slack, Maria E Giannakou, Andrea Foley, Martin Goss, Linda Partridge

**Affiliations:** 1 Institute of Healthy Ageing and GEE, University College LondonGower Street, London WC1E 6BT, UK

**Keywords:** *Drosophila*, aging, FOXO, insulin signaling

## Abstract

The insulin/insulin-like growth factor-like signaling (IIS) pathway in metazoans has evolutionarily conserved roles in growth control, metabolic homeostasis, stress responses, reproduction, and lifespan. Genetic manipulations that reduce IIS in the nematode worm *Caenorhabditis elegans*, the fruit fly *Drosophila melanogaster*, and the mouse have been shown not only to produce substantial increases in lifespan but also to ameliorate several age-related diseases. In *C. elegans*, the multitude of phenotypes produced by the reduction in IIS are all suppressed in the absence of the worm FOXO transcription factor, DAF-16, suggesting that they are all under common regulation. It is not yet clear in other animal models whether the activity of FOXOs mediate all of the physiological effects of reduced IIS, especially increased lifespan. We have addressed this issue by examining the effects of reduced IIS in the absence of dFOXO in *Drosophila*, using a newly generated null allele of *dfoxo*. We found that the removal of dFOXO almost completely blocks IIS-dependent lifespan extension. However, unlike in *C. elegans*, removal of dFOXO does not suppress the body size, fecundity, or oxidative stress resistance phenotypes of IIS-compromised flies. In contrast, IIS-dependent xenobiotic resistance is fully dependent on dFOXO activity. Our results therefore suggest that there is evolutionary divergence in the downstream mechanisms that mediate the effects of IIS. They also imply that in *Drosophila*, additional factors act alongside dFOXO to produce IIS-dependent responses in body size, fecundity, and oxidative stress resistance and that these phenotypes are not causal in IIS-mediated extension of lifespan.

## Introduction

The insulin/insulin-like growth factor (IGF)-like signaling (IIS) pathway of metazoans regulates such diverse processes as growth, developmental timing, body size, metabolism, stress responses, reproduction, and lifespan (Kenyon, 2005; Giannakou & Partridge, 2007). Genetic manipulations that inhibit IIS in the nematode worm *Caenorhabditis elegans*, the fruit fly *Drosophila melanogaster*, and the mouse not only increase lifespan but also delay the onset of age-related pathology and disease (Tatar *et al.*, 2003; Kenyon, 2005; Bonkowski *et al.*, 2006; Cohen *et al.*, 2006; Wessells & Bodmer, 2007; Selman *et al.*, 2008; Wessells *et al.*, 2009). Direct downstream targets of IIS in worms, flies, and mammals are the FOXO (Forkhead bOX-containing protein, subfamily O) proteins, a highly conserved family of transcription factors. Phosphorylation of FOXOs by the insulin-activated protein kinases PKB/AKT and SGK leads to their sequestration within the cytoplasm and, as a result, transcriptional inactivation of target gene expression (van der Horst & Burgering, 2007; Partridge & Bruning, 2008). Several direct FOXO target genes have been identified that function during cell cycle control, metabolism, apoptosis, and the regulation of cellular stress responses (Greer & Brunet, 2005, 2008; Partridge & Bruning, 2008; Salih & Brunet, 2008). Hence, the activation of FOXOs and their target genes has been under intense study to identify the transcriptional changes associated with IIS-dependent lifespan extension.

Lifespan extensions induced by decreasing the activity of the insulin/IGF1-like receptor, DAF-2, or downstream components of the IIS pathway in *C. elegans* are completely dependent upon the activity of the worm FOXO transcription factor, DAF-16 (Kenyon *et al.*, 1993). Thus, mutation of *daf-16* or reductions in its expression by RNAi can completely abrogate the lifespan extension observed in mutants for *daf-2*, the worm insulin/IGF receptor orthologue, or *age-1*, the worm phosphatidylinositol 3-kinase orthologue (Kenyon *et al.*, 1993). In other model organisms, FOXOs clearly play important roles during lifespan determination: overexpression of dFOXO protein in the adult fat body increases lifespan in *Drosophila* (Tatar *et al.*, 2003; Giannakou *et al.*, 2004; Hwangbo *et al.*, 2004), while heterozygous knockouts for the insulin receptor substrates, IRS1 or IRS2, are long lived and show increased activity of FOXO1 target genes in murine models (Taguchi *et al.*, 2007; Selman *et al.*, 2008). Furthermore, genetic variation in the *Foxo3A* gene is associated with longevity in several different human populations (Kuningas *et al.*, 2007; Willcox *et al.*, 2008; Flachsbart *et al.*, 2009). However, it has yet to be shown in these other animal models whether the effects of reduced IIS on lifespan are directly dependent on FOXO activity.

In addition to lifespan extension, decreasing IIS in the worm produces a number of other phenotypic responses that are all dependent upon DAF-16, suggesting that may all be regulated by a common mechanism. For example, *daf-2-*dependent reproductive delay and oxidative stress resistance are completely suppressed by knockdown of *daf-16* expression by RNAi (Larsen, 1993; Honda & Honda, 1999; Dillin *et al.*, 2002). Also, DAF-16 mediates both stress resistance and reduced adult fecundity in *age-1* mutants (Larsen, 1993; Tissenbaum & Ruvkun, 1998; Honda & Honda, 1999). dFOXO-dependent effects on IIS-mediated growth control and germline stem cell (GSC) proliferation have been reported in *Drosophila* (Junger *et al.*, 2003; Puig *et al.*, 2003; Hsu *et al.*, 2008). Nevertheless, in other animals, it remains unclear whether FOXOs mediate all of the phenotypic effects of reduced IIS and thus whether this feature of the signaling pathway has been conserved through evolution.

In this study, we have examined whether several IIS-dependent phenotypes, including increased lifespan, reduced fecundity, increased stress resistance, developmental delay, and growth inhibition, are dependent on dFOXO activity in *Drosophila*. We combined a newly generated null allele of *dfoxo* with several models of reduced IIS in *Drosophila* including ubiquitous expression of a kinase-dead, dominant negative version of the *Drosophila* insulin receptor both during development and specifically restricted to adulthood, late ablation of the dilp-producing median neurosecretary cells (MNCs) and adult-specific ubiquitous expression of a dominant negative form of PI3 kinase, all of which show phenotypes typical of reduced IIS. We found that mutation of *dfoxo* almost completely blocked lifespan extension in these IIS-compromised flies. However, removal of *dfoxo* failed to rescue IIS-mediated developmental delay, small body size, reduced egg laying, and resistance to paraquat. In contrast, increased resistance to the xenobiotic toxin, dichlorodiphenyltrichloroethane (DDT), was completely dependent on dFOXO activity. Our results show that, unlike in *C. elegans*, where all phenotypic traits produced by reduced IIS are DAF-16 dependent, additional factors besides dFOXO have evolved in *Drosophila* to mediate the full IIS response.

## Results

### Generation and characterization of a new *dfoxo* null allele

**Fig. 1:**
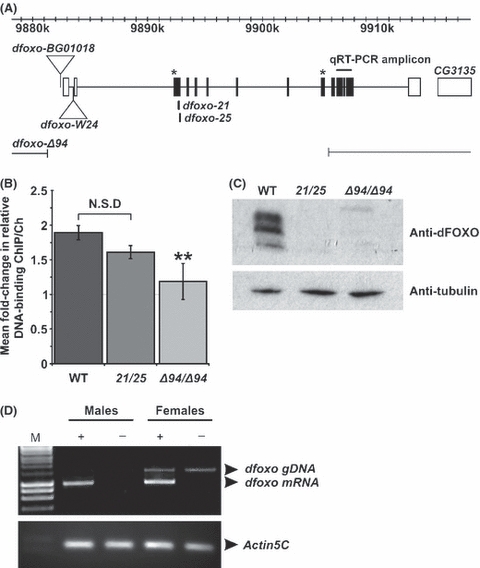
Molecular characterization of the *dfoxo*^*Δ94*^ deletion. (A) Schematic representation of the *dfoxo* locus. Coding exons are in black and noncoding exons are in white. The position of the P-element insertion (*dfoxo*^*BG01018*^*)* used to generate the *dfoxo*^*Δ94*^ deletion by imprecise excision and the breakpoints of the *dfoxo*^*Δ94*^ deletion are shown along with the positions of the *dfoxo*^*21*^, *dfoxo*^*25*^, and *dfoxo*^*w24*^ mutations. The position of the amplicon amplified by RT-PCR to detect *dfoxo* mRNA expression is also indicated along with the position of the sequences encoding the peptides from which the dFOXO antibody was generated (marked by *). (B) Quantitative PCR (qPCR) on the *Lnk* promoter normalized to a control genomic region located 3′ to the *Lnk* gene to determine the proportion of DNA recovered after chromatin immunoprecipitation (ChIP) using a specific anti-dFOXO antibody from chromatin extracts prepared from wild-type, *dfoxo*^*21*^*/dfoxo*^*25*^ and *dfoxo*^*Δ94*^ homozygous mutant flies. Relative DNA binding was calculated as the proportion of chromatin recovered in the ChIP divided by that in the total chromatin preparation. Data are presented as mean fold change in DNA binding at the *Lnk* 5′ region compared with the control *Lnk* 3′ region ± SEM of two (for *dfoxo*^*21*^*/dfoxo*^*25*^) or three biological repeats. (**P <0.05, *t*-test). (C) Western blot analysis of dFOXO protein expression in extracts prepared from wild-type, *dfoxo*^*21*^*/dfoxo*^*25*^ (*21/25*) heterozygotes and *dfoxo*^*Δ94*^ (*Δ94/Δ94*) homozygous mutant flies. Blots were probed with anti-tubulin as a control for protein loading. (D) RT-PCR analysis of *dfoxo* transcript expression in 7-day-old male and female wild-type (+) and *dfoxo*^*Δ94*^ homozygous mutant (*−*) flies. M indicates the marker lane. Amplification of the *actin5C* transcript was used as a control for the RT-PCR protocol.

Several loss-of-function mutants for the *Drosophila dfoxo* transcription factor have already been described. These include *dfoxo*^*21*^ and *dfoxo*^*25*^, both of which contain chemically induced nucleotide transversions within the *dfoxo* coding region, resulting in premature stop codons within the proposed DNA-binding domain of the protein (Junger *et al.*, 2003) and *dfoxo*^*W24*^, which contains a P-element insertion within the first intron of the *dfoxo* locus (Weber *et al.*, 2005; Fig. 1A). Heteroallelic combinations of these mutants produce viable adults but no detectable protein by western blot analysis and are therefore considered to function as genetic nulls (Junger *et al.*, 2003; Giannakou *et al.*, 2008; Min *et al.*, 2008). We have performed chromatin immunoprecipitation (ChIP) experiments on chromatin extracts prepared from *dfoxo*^*21*^*/dfoxo*^*25*^ transheterozygous flies using a specific dFOXO antibody, the epitope for which would still be present within any translated mutant protein (Fig. 1A). dFOXO DNA binding was assessed using a promoter region of the *Drosophila* SH2B-encoding gene, *Lnk*, which we have previously demonstrated to be bound by dFOXO (Slack *et al.*, 2010). Surprisingly, quantitative PCR (qPCR) after dFOXO ChIP from *dfoxo*^*21*^*/dfoxo*^*25*^ samples showed enrichment of the *Lnk* promoter fragment relative to a control genomic region, similar to wild-type controls (Fig. 1B). Thus, despite the apparent absence of dFOXO protein in *dfoxo*^*21*^*/dfoxo*^*25*^ mutants (Fig. 1C), there still appears to be residual DNA-binding activity in these flies. This allelic combination may therefore function more as a dominant negative rather than as a true null. Interestingly, dominant effects of the *dfoxo*^*21*^ allele have been observed in other studies (Nielsen *et al.*, 2008).

We therefore generated a new deletion mutant of *dfoxo* by imprecise excision of a P-element positioned upstream of the first noncoding exon of the *dfoxo* gene. This deletion (*dfoxo*^*Δ94*^*)* spans over 20 kb of the *dfoxo* locus, removing part of the predicted promoter region as well as several coding exons. Homozygotes for the deletion were adult viable, and neither dFOXO protein expression nor DNA-binding activity was detected in these flies (Fig. 1B,C). However, the deletion removes the sequence encoding the epitope site for the dFOXO antibody, and so we could not exclude the possibility that some mutant protein is produced. We therefore examined the expression of *dfoxo* mRNA by RT-PCR using primers that anneal outside of the deleted region and found that homozygous mutants were completely devoid of *dfoxo* transcript expression (Fig. 1D). Consequently, this deletion appears to represent a true null allele of *dfoxo*.

**Fig. 2:**
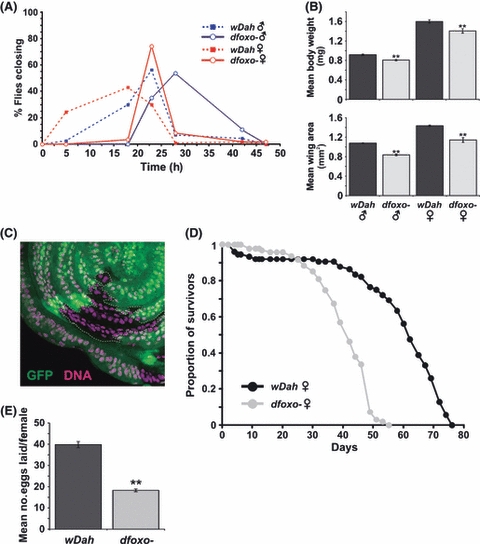
Phenotypic analysis of *dfoxo*^*Δ94*^ homozygous mutant flies. (A) Egg-to-adult development time is delayed in both males and females homozygous for the *dfoxo*^*Δ94*^ deletion (*dfoxo−*) compared with *wDahomey* controls. Only the eclosion period of the adult flies is shown. Data are shown as percentage of flies eclosing. (For males, *n* = 114 for *wDahomey* and *n* = 106 for *dfoxo−.* For females, *n* = 107 for *wDahomey* and *n* = 116 for *dfoxo−*). (B) Homozygous *dfoxo*^*Δ94*^ flies (*dfoxo−*) have significantly reduced adult body weights and smaller wing sizes than *wDahomey* controls. Data are represented as means ± SEM (*n* = 10 for each measurement, **P <0.05, anova). (C) Clonal analysis of the effects of *dfoxo*^*Δ94*^ mutation on cell size and proliferation in the developing wing disk. *dfoxo*^*Δ94*^ mutant cells were generated by mitotic recombination and are marked by the absence of GFP. No obvious differences were observed in clone size nor in the size of in *dfoxo*^*Δ94*^ mutant cells within the clone (GFP-negative cells) compared with adjacent heterozygous cells (GFP-positive cells) outside of the clone. (D) Survival curves of female flies homozygous mutant for *dfoxo−* mutants (*n* = 99, median survival = 41 days, maximum survival = 48 days) and *wDahomey* controls (*n* = 94, median survival = 61 days, maximum survival = 73 days). *dfoxo−* mutants are significantly shorter lived than controls (*P <*0.0001; Log-rank test). Representative of two independent experiments. (E) Average number of eggs laid per *dfoxo−* mutant 7-day-old female compared with age-matched *wDahomey* controls. Data are presented as the mean number of eggs laid per female over a 24-h period ± SEM. Eggs were counted from ten separate vials, and each vial contained ten females. *dfoxo−* females laid significantly fewer eggs than *wDahomey* controls. (**P <0.05, anova).

*dfoxo*^*Δ94*^ homozygotes were delayed in egg–adult development time and were also smaller in size than their controls, with significant reductions in both body weight and wing area (Fig. 2A,B). No obvious effects on developmental time or body weight have been previously reported for other *dfoxo* mutants, with only a small decrease in wing size described for *dfoxo*^*21*^*/dfoxo*^*25*^ transheterozygotes (Junger *et al.*, 2003). Nevertheless, delayed egg-adult development and reduced body size were also observed in transheterozygous *dfoxo*^*25*^*/dfoxo*^*Δ94*^ flies as well as in hemizygous *Df(3R)ED5624/dfoxo*^*Δ94*^ flies using a deficiency that removes the entire *dfoxo* locus (Supplementary Fig. S1), confirming that the observed effects on developmental time and body size in *dfoxo*^*Δ94*^ homozygotes are specific to the *dfoxo*^*Δ94*^ genetic lesion.

The small body size of *dfoxo*^*Δ94*^ homozygotes suggested that dFOXO activity could potentially regulate cell growth or proliferation. To examine this, we generated clones of *dfoxo*^*Δ94*^ mutant cells in an otherwise heterozygous animal by mitotic recombination. Both clone size and *dfoxo*^*Δ94*^ mutant cell size were normal (Fig. 2C), as has been observed with other *dfoxo* alleles (Junger *et al.*, 2003), suggesting that dFOXO does not act cell-autonomously to restrict cell proliferation or growth. Hence, the effects of the *dfoxo*^*Δ94*^ mutation on growth must occur via nonautonomous mechanisms. Similar to other *dfoxo* allelic combinations, homozygous *dfoxo*^*Δ94*^ females were shorter lived than their controls (Fig. 2B) and also laid fewer eggs (Fig. 2C).

The *dfoxo*^*Δ94*^ deletion was then combined with several mutations or genetic manipulations that reduce IIS in *Drosophila* including a dilp2-3,5 triple mutant (Gronke *et al.*, 2010), median neurosecretary cell (mNSC) ablation (Broughton *et al.*, 2005), *chico*^*1*^ mutants (Bohni *et al.*, 1999), and *Lnk*^*Del29*^ mutants (Slack *et al.*, 2010). All resulted in preadult lethality when combined with homozygosity for *dfoxo*^*Δ94*^. Interestingly, the lethality of *chico*^*1*^*; dfoxo*^*Δ94*^ homozygotes was rescued by the expression of a *UAS-dfoxo* transgene within the MNCs, suggesting that a dFOXO-dependent transcriptional response specifically within the MNCs is both necessary and sufficient for the viability of *chico*^*1*^ homozygotes.

Viable flies were obtained when the *dfoxo*^*Δ94*^ mutant was combined with either overexpression of a dominant negative form of the *Drosophila* insulin receptor (*UAS-InRDN*) under the control of the ubiquitous and constitutive *daughterless*-GAL4 driver (*daGAL*) or late ablation of the mNSCs by the expression of *UAS*-*reaper* (*UAS-rpr*) under the control of the *InsP3-GAL4* driver (*InsP3GAL*). In addition, we examined adult-onset ubiquitous expression of *UAS-InRDN* as well as adult-onset ubiquitous expression of a catalytically inactive, dominant negative form of PI3 kinase (*UAS-Dp110DN)* using the inducible *daughterless*-*GeneSwitch* (*daGS*) driver. *daGS* only drives transgene expression in the presence of the RU486 steroid drug. Treatment with RU486 had no effect on the lifespan, fecundity, or stress resistance of *daGS/+* flies themselves (Supplementary Fig. S2).

### IIS-mediated longevity requires dFOXO activity

**Fig. 3:**
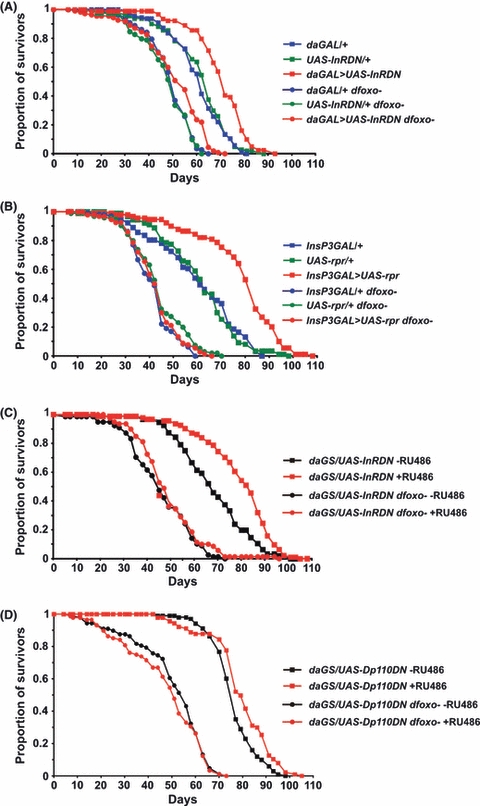
Effects of *dfoxo* removal on the survivorship of IIS-compromised flies. (A) Survival curves of female flies overexpressing a dominant negative version of the insulin receptor (*daGAL >* *UAS-InRDN*) and their genetic controls (*daGAL/+* and *UAS-InRDN/+*) in both wild-type and *dfoxo−* backgrounds (representative of two independent experiments). In a wild-type background: for *daGAL >* *UAS-InRDN* median survival = 71 days, maximum survival = 82 days, *n* = 93; for *daGAL/+* median survival = 61 days, maximum survival = 77 days, *n* = 114; for *UAS-InRDN/+* median survival = 64 days, maximum survival = 75, *n* = 115. The survival of *daGAL >* *UAS-InRDN* flies was significantly different from each of the controls (*P <*0.0001; log-rank test). No significant difference in survival was detected between the two controls (*P* = 0.6, log-rank test). In a *dfoxo−* background: for *daGAL >* *UAS-InRDN* median survival = 53 days, maximum survival = 64 days, *n* = 114; for *daGAL/+* median survival = 49 days, maximum survival = 59 days, *n* = 109; for *UAS-InRDN/+* median survival = 49 days, maximum survival = 57 days, *n* = 106. The survival of *daGAL >* *UAS-InRDN dfoxo−* flies is significantly different from each of the controls (*P <*0.001; log-rank test). No significant difference in survival was detected between the two controls (*P* = 0.5, log-rank test). (B) Survival curves of female flies with late ablation of the median neurosecretary cells (*InsP3GAL >* *UAS-rpr*) and their genetic controls (*InsP3GAL/+* and *UAS-rpr/+*) in both wild-type and *dfoxo−* backgrounds. In a wild-type background: for *InsP3GAL >* *UAS-rpr* median survival = 82 days, maximum survival = 100 days, *n* = 99; for *InsP3/+* median survival = 60 days, maximum survival = 82 days, *n* = 94; for *UAS-rpr/+* median survival = 62 days, maximum survival = 82 days, *n* = 93. The survival of *InsP3GAL >* *UAS-rpr* flies was significantly different from each of the controls (*P <*10^−12^, log-rank test). No significant difference in survival was detected between the two controls (*P* = 0.8, log-rank test). In a *dfoxo−* background: for *InsP3GAL >* *UAS-rpr* median survival = 44 days, maximum survival = 58 days, *n* = 94; for *InsP3/+* median survival = 41 days, maximum survival = 56 days, *n* = 108; for *UAS-rpr/+* median survival = 44 days, maximum survival = 59 days, *n* = 95. The survival of *InsP3GAL >* *UAS-rpr* flies was not significantly different from either of the controls (*P*>0.2, log-rank test). (C) Survival curves of female *daGS >* *UAS-InRDN* flies induced to ubiquitously express the dominant negative insulin receptor by feeding RU486-containing food from day 3 of adulthood (wild-type +RU486: median survival = 83 days, maximum survival = 95 days, *n* = 94; *dfoxo−* +RU486: median survival = 46 days, maximum survival = 70 days, *n* = 81) compared with uninduced controls (wild-type *−*RU486: median survival = 67 days, maximum survival = 90 days, *n* = 97; *dfoxo−−*RU486: median survival = 44 days, maximum survival = 65 days, *n* = 79). The survival of wild-type *daGS >* *UAS-InRDN* +RU486 was significantly different from the −RU486 control (*P <*10^−6^, log-rank test). The survival of *dfoxo− daGS >* *UAS-InRDN* +RU486 was not significantly different from the −RU486 control (*P* = 0.2, log-rank test). (D) Survival curves of female *daGS >* *UAS-dp110DN* flies induced to ubiquitously overexpress a dominant negative form of Dp110 by feeding RU486-containing food from day 3 of adulthood (wild-type +RU486: median survival = 80 days, maximum survival = 97 days, *n* = 91; *dfoxo−* +RU486: median survival = 51 days, maximum survival = 65 days, *n* = 95) compared with uninduced controls (wild-type *−*RU486: median survival = 75 days, maximum survival = 92 days, *n* = 103; *dfoxo−−*RU486: median survival = 54 days, maximum survival = 65 days, *n* = 97). The survival of wild-type *daGS >* *UAS-dp110DN* +RU486 was significantly different from the −RU486 control (*P*<0.001, log-rank test). The survival of *dfoxo− daGS >* *UAS-dp110DN* +RU486 was not significantly different from the −RU486 control (*P* = 0.4, log-rank test). Representative of two independent experiments.

In the presence of *dfoxo*, *daGAL > UAS-InRDN* females lived significantly longer than controls, with a 10–15% increase in median lifespan and 6–10% increase in maximum lifespan (Fig. 3A). In a *dfoxo−* background, all groups were shorter lived compared with their wild-type counterparts, yet *daGAL > UAS-InRDN dfoxo−* flies showed an age-related increase in survival compared with both genetic controls (*daGAL/+ dfoxo−* and *UAS-InRDN/+ dfoxo−*) (Fig. 3A). When all flies were considered in the analysis, *daGAL > UAS-InRDN dfoxo−* flies were significantly longer lived than both genetic controls (*P <*0.001; log-rank test). However, no significant differences in survival were apparent between groups during the first 50 days of the experiment (*P* = 0.55; log-rank test), whereas survival at ages beyond 50 days was increased in *daGAL > UAS-InRDN dfoxo−* flies compared with both controls (*P <*0.0001; log-rank test) (Fig. 3A). Hence, maximum lifespan, calculated as the median age of the oldest 10% of the population to die, was significantly increased by approximately 8% (*P <*0.0001; log-rank) for *daGAL > UAS-InRDN dfoxo−* flies (66 days, *n* = 14) relative to both genetic controls (54 days, *n* = 11 for *daGAL/+ dfoxo−* flies and 59 days, *n* = 16 for *UAS-InR-DN dfoxo−* flies). This apparent age-related difference in survival was observed in a second, independent experiment, suggesting that ubiquitous and constitutive expression of *UAS-InRDN* can increase survival later in life and hence extend maximum lifespan even in the absence of dFOXO activity.

*InsP3GAL > UAS-rpr* females also lived significantly longer than both genetic controls (*InsP3GAL/+* and *UAS-rpr/+*) in the presence of *dfoxo* with approximately 30% increase in median and approximately 20% in maximum lifespan (Fig. 3B). Again, all groups were shorter lived in a *dfoxo−* background, but in contrast to *daGAL > UAS-InRDN* flies, *InsP3GAL > UAS-rpr* were not significantly longer lived than their genetic controls in a *dfoxo−* background (Fig. 3B).

In both *daGS > UAS-InRDN* and *daGS > UAS-Dp110DN* flies, induction of transgene expression by RU486 increased lifespan in both genotypes, with median lifespan increased by 24% and 7%, respectively, and maximum lifespan increased by 5% for both genotypes compared with uninduced flies of the same genotypes (Fig. 3C,D). No extension of lifespan was observed in either *daGS > UAS-InRDN* and *daGS > UAS-Dp110DN* flies in a *dfoxo−* background upon RU486-induced transgene expression compared with uninduced controls (Fig. 3C,D).

Taken together, these data show that loss of dFOXO activity is sufficient to almost completely inhibit the longevity of IIS mutants in *Drosophila*. By contrast, treatment of *dfoxo−* females with the TOR kinase inhibitor, rapamycin, significantly increased median lifespan by approximately 10%, as was observed in rapamycin-treated *wDahomey* control females (Supplementary Fig. S3). Furthermore, *dfoxo−* females also showed an increase in lifespan under dietary restriction (Supplementary Fig. S4 and Table S1). Thus, loss of dFOXO activity specifically abrogates the increase in lifespan from reduced IIS.

### Loss of dFOXO activity fails to rescue IIS-mediated fecundity defects

IIS plays a complex role during oogenesis in *Drosophila*: autonomous IIS within the germline directly controls germline cyst development, vitellogenesis, and the rate of GSC divisions (LaFever & Drummond-Barbosa, 2005; Hsu *et al.*, 2008; Hsu & Drummond-Barbosa, 2009), while IIS via dilps indirectly controls the proliferation of the follicle cells (LaFever & Drummond-Barbosa, 2005). We therefore examined the effects of *dfoxo* removal on egg laying in IIS-compromised females.

**Fig. 4:**
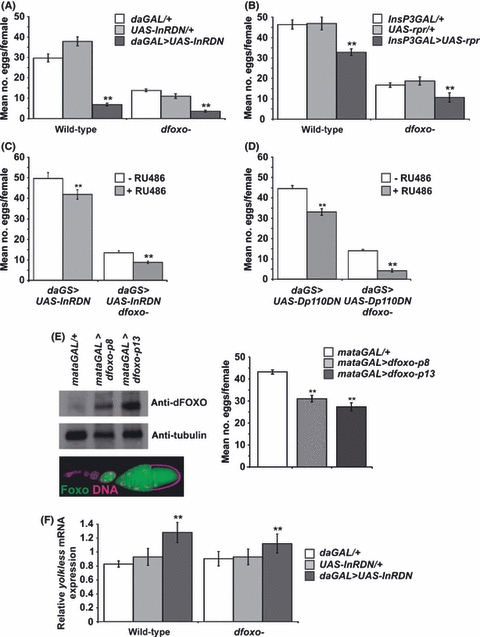
Effects of *dfoxo* removal on the female fecundity. (A–D) Average number of eggs laid per 7-day-old female or after 7 days of RU486 treatment. Data are presented as the mean number of eggs laid per female over a 24-h period ± SEM. Eggs were counted from ten separate vials, and each vial contained ten females. (A) Females with constitutive and ubiquitous expression of the dominant negative insulin receptor. In both wild-type and *dfoxo−* backgrounds, *daGAL >* *UAS-InRDN* females laid significantly fewer eggs than both *daGAL/+* and *UAS-InRDN/+* controls (**P <0.05, *t*-test). There was no significant difference between *daGAL >* *UAS-InRDN* and *daGAL >* *UAS-InRDN dfoxo−* flies. (B) Females with late ablation of the median neurosecretary cells. In both wild-type and *dfoxo−* backgrounds, *InsP3GAL >* *UAS-rpr* laid significantly fewer eggs than both *InsP3GAL/+* and *UAS-rpr/+* controls (**P <0.05, *t*-test). (C) Females induced to ubiquitously overexpress the dominant negative insulin receptor by feeding RU486-containing food from day 3 of adulthood. In both wild-type and *dfoxo−* backgrounds, *daGS >* *UAS-InRDN* females induced with RU486 (+RU486) laid significantly fewer eggs uninduced controls (−RU486) (**P <0.05, *t*-test). (D) Females induced to ubiquitously overexpress dominant negative Dp110 by feeding RU486-containing food from day 3 of adulthood. In both wild-type and *dfoxo−* backgrounds, *daGS >* *UAS-Dp110DN* females induced with RU486 (+RU486) laid significantly fewer eggs uninduced controls (−RU486) (**P <0.05, *t*-test). (E) dFOXO protein expression in the germline. Western blot analysis of dFOXO protein expression in ovaries overexpressing two independent dFOXO transgenes (*dfoxo-p8* and *dfoxo-p13*) within the germline under the control of the *mataGAL4* driver. Blots were probed with anti-tubulin as a control for protein loading. Ovaries from these females overexpressing dFOXO protein in the germline look structurally wild-type, but egg production is significantly reduced. Eggs were collected from 7-day-old females over a 24-h period and counted. Data are presented as the mean number of eggs laid per female over these 24-h periods ± SEM (**P <0.05, anova). (F) Quantitative RT-PCR analysis of *yolkless* mRNA expression in female flies of the indicated genotypes normalized to *actin5C*. Data are presented as means ± SEM (*n* = 5). *yolkless* expression is upregulated in *daGAL >* *UAS-InRDN* females compared with controls (**P <0.05, *t*-test) in both wild-type and *dfoxo−* backgrounds.

*daGAL4 > UAS-InRDN* females showed reduced egg laying compared with both *daGAL/+* and *UAS-InRDN/+* controls in both wild-type and *dfoxo−* genetic backgrounds (Fig. 4A). Similar results were observed for *InsP3GAL > UAS-rpr* females: egg laying was reduced compared with both *InsP3GAL/+* and *UAS-rpr/+* controls in both wild-type and *dfoxo−* backgrounds (Fig. 4B). Ubiquitous adult-specific induction of *UAS-InRDN* or *UAS-Dp110DN* expression also decreased female fecundity and, again, this decrease in female egg laying was still observed in a *dfoxo−* background (Fig, 4C,D). Taken together, these data show that removal of *dfoxo* is not sufficient to rescue the reduced fecundity of IIS-compromised females. Furthermore, in a *dfoxo−* mutant background, *InsP3GAL > UAS-rpr* females laid significantly fewer eggs than their *dfoxo−* genetic controls (*P <*0.05, *t*-test) and both *daGS > InRDN dfoxo−* and *daGS > Dp110DN dfoxo−* females treated with RU486 laid significantly fewer eggs than their uninduced controls (*P <*0.001, anova), suggesting that loss of dFOXO activity and reduced IIS in these flies acted additively to reduce egg laying.

Mutation of *dfoxo* can reverse the reduction in GSC proliferation caused by the loss of *chico* function, demonstrating that dFOXO is required in the GSCs for at least some of the effects of lowered IIS on reproduction (Hsu *et al.*, 2008). To further examine the effects of dFOXO activity more specifically within the germline, we generated flies overexpressing a dFOXO transgene in the GSCs, using the maternal GAL4 driver, *mata-GAL4*. Despite significant overexpression of dFOXO protein in the ovaries of these females, they were still fertile with no overall gross morphological defects in ovarian structure (Fig. 4E). However, egg laying by these females was reduced by approximately 30% (Fig. 4E), demonstrating that increased dFOXO activity specifically within the germline is sufficient to reduce egg production.

In *daGAL4 > UAS-InRDN* females, the dominant negative insulin receptor is expressed in all somatic cells but not in the germline. Therefore, the reduction in egg laying in these females must be mediated via indirect responses of the germline to somatic signals. IIS can indirectly affect egg production through the regulation of yolk protein uptake into the oocycte during vitellogenesis (Richard *et al.*, 2005). It is therefore possible that dFOXO transcriptional activity is required for the expression of yolk protein transcripts themselves. However, we found no significant difference in the expression of *yolk protein 2 (YP2)* in *daGAL4 > UAS-InRDN* females, in either a wild-type or *dfoxo−* background, indicating that *YP2* expression is unresponsive to both IIS itself and dFOXO activity. In contrast, transcription of the yolk protein receptor, *yolkless*, whose expression is normally restricted to the oocyte, was significantly increased in *daGAL4 > UAS-InRDN* females compared with controls (Fig. 4F). However, this increase in *yolkless* expression was still present in *dfoxo−* mutants (Fig. 4F) and so occurs independently of dFOXO-mediated transcriptional regulation.

### dFOXO is not required for IIS-mediated oxidative stress resistance

**Fig. 5:**
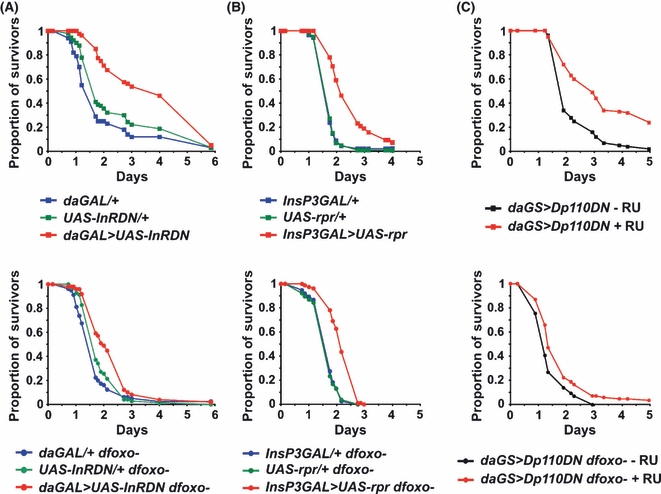
Functions for dFOXO during IIS-mediated oxidative stress resistance. (A) Survival curves in response to 20 mm paraquat of female flies overexpressing a dominant negative version of the insulin receptor (*daGAL >* *UAS-InRDN*) and their genetic controls (*daGAL/+* and *UAS-InRDN/+*) in both wild-type and *dfoxo−* backgrounds (representative of two independent experiments). In a wild-type background (top panel): for *daGAL >* *UAS-InRDN* median survival = 3.5 days, maximum survival = 4.9 days, *n* = 100; for *daGAL/+* median survival = 1.5 days, maximum survival = 1.5 days, *n* = 90; for *UAS-InRDN/+* median survival = 1.5 days, maximum survival = 4.9, *n* = .80. The survival of *daGAL >* *UAS-InRDN* flies was significantly different from each of the controls (*P <*10^−6^; Log-rank test). In a *dfoxo−* background (bottom panel): for *daGAL >* *UAS-InRDN* median survival = 1.9 days, maximum survival = 3.5 days, *n* = 49; for *daGAL/+* median survival = 1.5 days, maximum survival = 2.9 days, *n* = 80; for *UAS-InRDN/+* median survival = 1.5 days, maximum survival = 2.4 days, *n* = 70. The survival of *daGAL >* *UAS-InRDN dfoxo−* flies was significantly different from each of the controls (*P <*0.05; log-rank test). (B) Survival curves in response to 20 mm paraquat of female flies with late ablation of the median neurosecretary cells (*InsP3GAL >* *UAS-rpr*) and their genetic controls (*InsP3GAL/+* and *UAS-rpr/+*) in both wild-type and *dfoxo−* backgrounds. In a wild-type background (top panel): for *InsP3GAL >* *UAS-rpr* median survival = 2.1 days, maximum survival = 3.9 days, *n* = 95; for *InsP3GAL/+* median survival = 1.5 days, maximum survival = 2.1 days, *n* = 88; for *UAS-rpr/+* median survival = 2.1 days, maximum survival = 1.9, *n* = 99. The survival of *InsP3GAL >* *UAS-rpr* flies was significantly different from each of the controls (*P <*10^−15^; log-rank test). In a *dfoxo−* background (bottom panel): for *InsP3GAL >* *UAS-rpr* median survival = 2.1 days, maximum survival = 2.5 days, *n* = 78; for *InsP3GAL/+* median survival = 1.5 days, maximum survival = 2.1 days, *n* = 77; for *UAS-Rpr/+* median survival = 1.5 days, maximum survival = 2.1 days, *n* = 76. The survival of *InsP3GAL >* *UAS-rpr dfoxo−* flies was significantly different from each of the controls (*P <*10^*−*12^; log-rank test). (C) Survival curves in response to 20 mm paraquat of female flies induced to overexpress dominant negative Dp110 (*daGS >* *UAS-Dp110DN*) using RU486 (+RU486) and their uninduced controls (−RU486) in both wild-type and *dfoxo−* backgrounds. In a wild-type background (top panel): for *+*RU486 median survival = 2.6 days, maximum survival = 6.5 days, *n* = 100; for *−*RU486 median survival = 1.6 days, maximum survival = 3.6 days, *n* = 100. The survival of *+*RU486 flies was significantly different from the uninduced controls (*P <*10^−9^; log-rank test). In a *dfoxo−* background (bottom panel): for *+*RU486 median survival = 1.3 days, maximum survival = 2.6 days, *n* = 85; for *−*RU486 median survival = 1.1 days, maximum survival = 2.2 days, *n* = 0.84. The survival of *+*RU486 flies was significantly different from the uninduced controls (*P <*0.001); log-rank test).

Genetic interventions that inhibit IIS often result in enhanced resistance to various stresses including oxidative stress (Clancy *et al.*, 2001; Broughton *et al.*, 2005). We therefore examined the effects of paraquat, an intracellular ROS generator, on the survival of *daGAL4 > UAS-InRDN*, *InsP3GAL > UAS-rpr* and *daGS > Dp110DN* flies. In a wild-type background, *daGAL4 > UAS-InRDN, InsP3GAL > UAS-rpr* and *daGS > Dp110DN* flies all survived for significantly longer on food supplemented with 20 mm paraquat compared with their respective controls (Fig. 5A,B,C). Interestingly, we still observed a small but significant and proportionally similar increase in the survival of *daGAL4 > UAS-InRDN dfoxo−*, *InsP3GAL > UAS-rpr dfoxo−* and *daGS > Dp110DN dfoxo−* flies over their respective controls (Fig. 5A,BC), showing that in the absence of dFOXO activity, reductions in IIS can still increase resistance to paraquat treatment.

### IIS-dependent xenobiotic metabolism is dFOXO-dependent

**Fig. 6:**
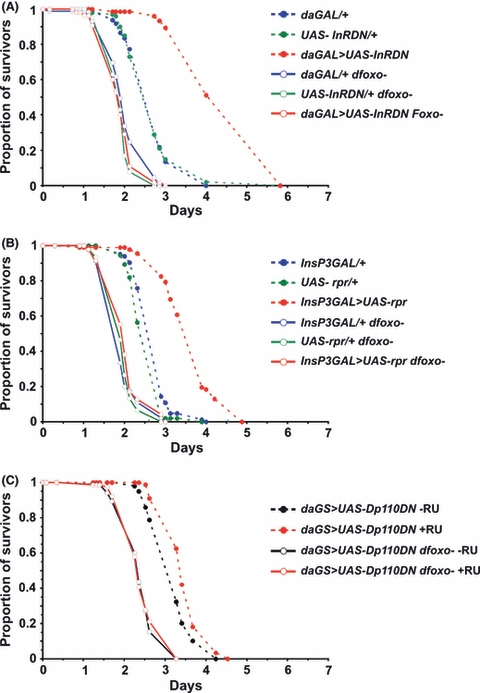
Functions for dFOXO during IIS-mediated dichlorodiphenyltrichloroethane (DDT) resistance. (A) Survival curves in response to DDT of female flies overexpressing a dominant negative version of the insulin receptor (*daGAL >* *UAS-InRDN*) and their genetic controls (*daGAL/+* and *UAS-InRDN/+*) in both wild-type and *dfoxo−* backgrounds. In a wild-type background: for *daGAL >* *UAS-InRDN* median survival = 4.9 days, maximum survival = 4.9 days, *n* = 76; for *daGAL/+* median survival = 2.4 days, maximum survival = 3.5 days, *n* = 97; for *UAS-InRDN/+* median survival = 2.4 days, maximum survival = 3.5, *n* = 100. The survival of *daGAL >* *UAS-InRDN* flies was significantly different from each of the controls (*P <*10^−24^; log-rank test). In a *dfoxo−* background: for *daGAL >* *UAS-InRDN* median survival = 1.8 days, maximum survival = 2.4 days, *n* = 55; for *daGAL/+* median survival = 1.8 days, maximum survival = 2.4 days, *n* = 89; for *UAS-InRDN/+* median survival = 1.8 days, maximum survival = 2.1 days, *n* = 65. The survival of *daGAL >* *UAS-InRDN dfoxo−* flies was not significantly different from each of the controls (*P*>0.1; log-rank test). (B) Survival curves in response to DDT of female flies with late ablation of the median neurosecretary cells (*InsP3GAL > UAS-rpr*) and their genetic controls (*InsP3GAL/+* and *UAS-rpr/+*) in both wild-type and *dfoxo−* backgrounds. In a wild-type background: for *InsP3GAL > UAS-rpr* median survival = 3.6 days, maximum survival = 4.5 days, *n* = 92; for *InsP3GAL/+* median survival = 2.6 days, maximum survival = 3.1 days, *n* = 83; for *UAS-rpr/+* median survival = 2.6 days, maximum survival = 2.6 days, *n* = 94. In a *dfoxo−* background: for *InsP3GAL > UAS-rpr* median survival = 1.9 days, maximum survival = 2.6 days, *n* = 90; for *InsP3GAL/+* median survival = 1.6 days, maximum survival = 2.6 days, *n* = 90; for *UAS-Rpr/+* median survival = 1.6 days, maximum survival = 2.3 days, *n* = 94. The survival of *InsP3GAL > UAS-rpr dfoxo−* flies was not significantly different from each of the controls (*P >*0.08; log-rank test). (C) Survival curves in response to DDT of female flies induced to overexpress dominant negative Dp110 (*daGS > UAS-Dp110DN*) using RU486 (+RU486) and their uninduced controls (−RU486) in both wild-type and *dfoxo−* backgrounds. In a wild-type background (top panel): for *+*RU486 median survival = 3.3 days, maximum survival = 4.0 days, *n* = 88; for *−*RU486 median survival = 2.9 days, maximum survival = 4.0 days, *n* = 99. The survival of *+*RU486 flies was significantly different from the uninduced controls (*P <*0.001; log-rank test). In a *dfoxo−* background (bottom panel): for *+*RU486 median survival = 2.3 days, maximum survival = 2.9 days, *n* = 76; for *−*RU486 median survival = 2.3 days, maximum survival = 2.9 days, *n* = 85. The survival of *+*RU486 flies was not significantly different from the uninduced controls (*P* = 0.6; log-rank test).

In both worms and flies, long-lived IIS mutants show increased expression of genes involved in xenobiotic metabolism (McElwee *et al.*, 2007), and IIS mutants in *Drosophila* show increased survival in the presence of the xenobiotic toxin, DDT (Gronke *et al.*, 2010). In a wild-type background, *daGAL4 > UAS-InRDN*, *InsP3GAL > UAS-rpr* and *daGS > Dp110DN* flies survived for longer in the presence of DDT compared with their controls (Fig. 6A,B,C). In a *dfoxo−* background, all experimental and control groups showed increased sensitivity to DDT and unlike with paraquat treatment, *daGAL4 > UAS-InRDN dfoxo−*, *InsP3GAL > UAS-rpr dfoxo−* and *daGS > Dp110DN dfoxo−* flies did not show any increased resistance to DDT over their respective controls (Fig. 6A,B,C). Thus, removal of dFOXO activity not only increased sensitivity to DDT treatment but completely abrogated the increased resistance to DDT of IIS-compromised flies.

### dFOXO-independent effects on developmental delay and growth

**Fig. 7:**
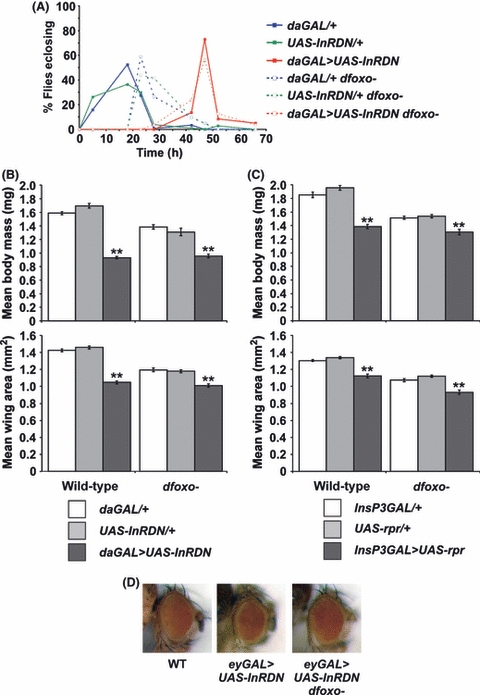
Effects of dFOXO removal on IIS-mediated developmental delay and growth inhibition. (A) Egg-to-adult development time is delayed in females overexpressing the dominant negative insulin receptor (*daGAL > UAS-InRDN*) irrespective of the presence or absence of *dfoxo*. Only the eclosion period of the adult flies is shown. Data are shown as percentage of flies eclosing. (*n* = 118 for *daGAL > UAS-InRDN*, *n* = 110 for *daGAL > UAS-InRDN dfoxo−*; *n* = 120 for *daGAL/+*, *n* = 102 for *daGAL/+ dfoxo−*; *n* = 107 for *UAS-InRDN/+*, *n* = 82 for *UAS-InRDN/+ dfoxo−*). (B) Body weights and wing areas as indicators of adult fly body size in females overexpressing the dominant negative insulin receptor (*daGAL > UAS-InRDN*). Data are presented as means ± SEM (*n* = 10 for each measurement). *daGAL > UAS-InRDN* females in both wild-type and *dfoxo−* backgrounds are significantly reduced in body size compared with control flies (*daGAL/+* and *UAS-InRDN/+*) (**P <0.05, *t*-test). (C) Body weights and wing areas as indicators of adult fly body size in females with late ablation of the median neurosecretary cells (*InsP3GAL > UAS-rpr*). Data are presented as means ± SEM (*n* = 10 for each measurement). *InsP3GAL > UAS-rpr* females in both wild-type and *dfoxo−* backgrounds are significantly reduced in body size compared with control flies (*InsP3GAL/+* and *UAS-rpr/+*) (**P <0.05, *t*-test). (D) Removal of dFOXO rescued the growth inhibition effects of overexpressing the dominant negative insulin receptor in a tissue-restricted manner. *UAS-InRDN* was specifically expressed in the developing eye using *eyGAL*. This resulted in a smaller eye compared with control (middle panel compared with left panel). This tissue-restricted growth inhibition was fully rescued in homozygous *dfoxo*^*Δ94*^ (*dfoxo−*) mutant flies (right panel).

*daGAL > UAS-InRDN* females were delayed in egg–adult development time by over 24 h compared with both the *daGAL/+* and *UAS-InRDN/+* controls (Fig. 7A). They also showed a significant reduction in both body weight and wing size (Fig. 7B). Removal of *dfoxo* did not rescue either the developmental delay or reduced body size of *daGAL > UAS-InRDN* females (Fig. 7A,B). *InsP3GAL > UAS-rpr* females were not delayed in their development time but were significantly smaller than both *InsP3/+* and *Uas-rpr/+* controls (Fig. 7C). Again, *InsP3GAL > UAS-rpr* females were still significantly smaller than controls in a *dfoxo−* mutant background (Fig. 7C). Thus, global removal of *dfoxo* is not sufficient to rescue the small body size of IIS mutant flies. Interestingly, when we restricted the expression of the dominant negative insulin receptor to the developing eye using *eyGAL4* (which produces a smaller eye under wild-type conditions), we observed a complete rescue of growth inhibition in the *dfoxo*^*Δ94*^ mutant background (Fig. 7D). It is therefore possible that dFOXO activity is required for the production of systemic growth factors that are required for proper organismal growth.

**Fig. 8:**
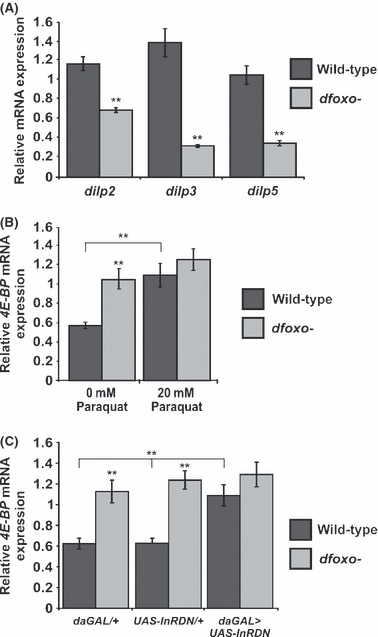
Gene expression changes in the absence of dFOXO activity. (A) Quantitative RT-PCR analysis of *dilp-2, dilp-3*, and *dilp-5* mRNA expression in female heads isolated from flies of the indicated genotypes normalized to *actin5C*. Expression of all three *dilp* transcripts is significantly decreased in *dfoxo−* flies compared with controls. Data are presented as means ± SEM (*n* = 5; *P <*0.05, *t*-test). (B) Quantitative RT-PCR analysis of *4E-BP* mRNA expression normalized to *actin5C* in female flies treated with 20 mm paraquat. In wild-type flies, *4E-BP* mRNA expression was significantly upregulated in response to paraquat treatment. In *dfoxo*^*Δ94*^ homozygous mutant flies, *4E-BP* expression was upregulated in comparison with wild-type flies but no further increase in *4E-BP* expression was observed when *dfoxo*^*Δ94*^ mutants were treated with paraquat. Data are presented as means ± SEM (*n* = 5; *P <*0.05, *t*-test). (C) Quantitative RT-PCR analysis of *4E-BP* mRNA expression in female flies of the indicated genotypes normalized to *actin5C*. In a wild-type background, *4E-BP* mRNA expression was significantly upregulated in *daGAL > UAS-InRDN* females compared with each of the genetic controls (*P <*0.05, *t*-test). In *dfoxo*^*Δ94*^ homozygous mutants, *4E-BP* mRNA expression is upregulated in both controls and experimental flies (*P <*0.05, *t*-test). However, no further increases in expression were detected in *daGAL > UAS-InRDN* in a *dfoxo* mutant background over wild-type background.

Candidates for systemic growth factors regulated by dFOXO are the *Drosophila* insulin-like peptides or dilps. Three of these peptides (*dilp-2, dilp-3,* and *dilp*-*5*) are expressed in the MNCs of the *Drosophila* brain. Ablation of the MNCs or genetic deletion of all three MNC-expressed *dilps* produces small flies owing to systemic effects on IIS-mediated growth (Ikeya *et al.*, 2002; Broughton *et al.*, 2005; Gronke *et al.*, 2010). Furthermore, expression of *dilp3* has been shown to be dependent on dFOXO activity (Broughton *et al.*, 2008). In *dfoxo*^*Δ94*^ mutants, the expression of all three MNC-expressed *dilps* was significantly reduced compared with control flies (Fig. 8A).

### Effects of *dfoxo* deletion on dFOXO target gene expression

The translational regulator 4E-BP (encoded by *Thor*) has been well documented as a direct target of dFOXO (Junger *et al.*, 2003; Puig *et al.*, 2003). *4E-BP* expression is upregulated when dFOXO is activated either in response to low IIS or upon exposure to stressors such as paraquat. We therefore examined the effects of the *dfoxo*^*Δ94*^ mutation on *4E-BP* expression under various conditions in which dFOXO activity would normally be induced. Thus, *4E-BP* expression was upregulated in both *daGAL4 > UAS-InRDN* flies and wild-type flies exposed to 20 mm paraquat (Fig. 8B,C). Surprisingly, *4E-BP* expression was also increased to a comparable level in *dfoxo*^*Δ94*^ homozygous mutants themselves. However, in *dfoxo*^*Δ94*^ homozygotes, no further increases in *4E-BP* expression were observed in *daGAL4 > UAS-InRDN* flies or upon exposure to paraquat (Fig. 8B,C).

## Discussion

As a result of the pleiotropic effects of IIS on animal physiology, extension of lifespan by reduced IIS is often accompanied by other phenotypic responses, including reduced or delayed reproduction, growth inhibition, increased stress resistance, and metabolic dysregulation. In *C. elegans*, all of the phenotypic outcomes of reduced IIS are under a common regulatory mechanism, because they are all dependent on the transcriptional activity of the FOXO transcription factor, DAF-16 (Kenyon *et al.*, 1993; Dillin *et al.*, 2002). In *Drosophila* and mammals, many of the same physiological traits are affected by reduced IIS, but a requirement for FOXO transcriptional activity in mediating all of the phenotypic responses to reduced IIS, especially lifespan extension, in these other animal models is less well understood. In this study, we have combined a novel deletion mutant of *dfoxo* that is devoid of *dfoxo* mRNA expression with several models of reduced IIS in *Drosophila* to investigate the consequences of *dfoxo* removal on lifespan, fecundity, development, growth, and stress resistance.

In *C. elegans*, IIS-mediated lifespan extension is entirely dependent on the activity of DAF-16, and genetic manipulations that reduce IIS cannot extend the lifespan of *daf-16* mutant or RNAi-treated worms (Kenyon *et al.*, 1993; Dillin *et al.*, 2002). We have observed similar results in *Drosophila* in that mutation of *dfoxo* completely blocked the lifespan extension associated with late ablation of the MNCs as well as adult-specific expression of either a dominant negative form of the *Drosophila* insulin receptor or a dominant negative form of Dp110. However, we did observe an age-specific increase in survival late in life in *dfoxo* mutants with ubiquitous expression of the dominant negative insulin receptor during development. In these flies, no differences in survival were observed until after the flies were aged 50 days, after which experimental flies consistently out-lived controls, resulting in a significant extension of their maximum lifespan. While these effects on survival were reproduced in independent replicate experiments, they were not observed with any other IIS manipulation, suggesting that developmental expression of the dominant negative insulin receptor may produce effects that are not necessarily linked to reduced IIS. It is, however, intriguing to note that at least one genetic manipulation that reduces IIS in *Drosophila* can still increase lifespan in the absence of dFOXO activity. In contrast to its effects in IIS-mediated lifespan extension, *dfoxo* mutants still showed increased survival in response to treatment with rapamycin, a specific TOR kinase inhibitor, and in response to dietary restriction. Thus, the inhibition of lifespan extension by the removal of *dfoxo* appears to be specific to the downregulation of IIS.

In worms, DAF-16 is also required to mediate the reduction in brood size associated with the genetic perturbation of *daf-2* or *age-1* expression (Tissenbaum & Ruvkun, 1998). However, we have found that in *Drosophila*, removal of dFOXO activity fails to rescue the reduction in egg laying associated with reduced IIS and consequently the reduced fecundity of IIS-compromised females does not appear to be dFOXO-dependent. In fact, low IIS and removal of dFOXO activity actually had additive effects, causing further reductions in egg laying than reduced IIS alone. The nature of the genetic manipulations used in our study to reduce IIS exclude direct reductions within the germline itself, and so the observed effects on female fecundity must be mediated by disruption of somatic signals to the germline. Our data would suggest that these somatic signals act independently of dFOXO. In support of this, we observed dFOXO-independent effects on the expression of the vitellogenic gene, *yolkless*, with reduced somatic IIS. Our study also highlights important differences between worms and flies in the timing requirements for IIS during reproduction. In *C. elegans*, *daf-2* RNAi initiated at egg hatching caused a delay in reproduction, whereas *daf-2* RNAi during adulthood had no effect (Dillin *et al.*, 2002). Our data have shown that reductions to IIS specifically during adulthood by expression of the dominant negative insulin receptor or dominant negative Dp110 are sufficient to reduce female fecundity.

A role for dFOXO within the germline itself during oogenesis, however, cannot be excluded. Previous studies have shown that the effects of low IIS on GSC proliferation can be reverted by a reduction in dFOXO activity, suggesting that at least some aspects of oogenesis are regulated by dFOXO (Hsu *et al.*, 2008), and we have shown here that overexpression of dFOXO alone specifically within the germline is sufficient to reduce egg laying. Hence, dFOXO-dependent and dFOXO-independent processes may mediate the full effects of reduced IIS on oogenesis. The proliferation of the GSCs in response to diet has been shown to be regulated via both dFOXO-dependent and dFOXO-independent processes (Hsu *et al.*, 2008). Moreover, the Ras-binding domain of *Drosophila* PI3K is required for maximal PI3K activity during egg laying (Orme *et al.*, 2006). Therefore, signaling via both dFOXO and Ras/Mapk may together mediate the full IIS response during oogenesis. Removal of dFOXO alone would hence be insufficient to rescue IIS-mediated defects in egg laying.

In *C. elegans*, DAF-16 regulates the expression of several oxidative stress responsive genes such as *sod 3*, *mtl-1*, *ctl-1*, and *ctl-2* (Honda & Honda, 1999; Murphy *et al.*, 2003). Furthermore, oxidative stress resistance in *daf-2* mutant worms is entirely dependent on the presence of DAF-16 (Honda & Honda, 1999). In contrast, we have found that all of the models of reduced IIS tested in this study showed increased resistance to the intracellular ROS generator, paraquat, even in the absence of dFOXO. dFOXO-independent effects may therefore contribute, at least in part, to the increased survival of IIS-compromised flies in response to paraquat treatment. Thus, IIS-dependent oxidative stress resistance can be uncoupled from IIS-dependent lifespan extension based on their requirements for dFOXO, suggesting that they are mediated via nonoverlapping mechanisms. Hence, oxidative stress resistance is not causal in IIS-mediated lifespan extension. By comparison, increased resistance to the xenobiotic toxin, DDT, was completely abolished in *dfoxo* mutants, suggesting that it is entirely dependent upon dFOXO activity. Furthermore, *dfoxo* mutants were more sensitive to DDT treatment than wild-type controls, indicating that dFOXO activity is required for survival in the presence of DDT. Our findings that IIS-dependent DDT resistance and lifespan extension require dFOXO suggests that enhanced xenobiotic metabolism may contribute to longevity in long-lived IIS mutant flies. Interestingly, transcriptome analyses of IIS mutants from worms, flies, and mammals have shown that the regulation of cellular detoxification is an evolutionary conserved function of long-lived IIS mutants in all three model organisms (McElwee *et al.*, 2007).

Perhaps our most surprising observation was that in combination with several IIS mutants, removal of dFOXO caused developmental lethality, for example, *chico; foxo* double mutants were lethal at prepupal stages. Furthermore, we were able to rescue the lethality of *chico; foxo* double mutants by the expression of dFOXO within the MNCs. Thus, a dFOXO-dependent transcriptional response specifically within the MNCs is required for the viability of *chico* mutants. The MNCs express the *Drosophila* insulin-like peptides *dilp-2, dilp-3*, and *dilp-5*, and we have shown that dFOXO is required for the basal expression of all three MNC-expressed *dilps* because their expression is reduced in *dfoxo* mutant heads. This raises the possibility that dFOXO activity itself may regulate systemic IIS, supported by our observations that removal of dFOXO activity has nonautonomous effects on growth. Nonautonomous inhibition of both somatic and GSC divisions has also been reported for other *dfoxo* mutants (Junger *et al.*, 2003; Hsu *et al.*, 2008).

In *C. elegans*, the group of genes identified as direct targets of DAF-16 and that are differentially expressed in response to IIS are enriched for IIS pathway genes, suggesting that when IIS is low, DAF-16 increases insulin sensitivity by upregulating the expression of IIS pathway genes (Schuster *et al.*, 2010). It is probable that a similar feedback mechanism operates in *Drosophila*, mediated by dFOXO transcriptional regulation. In a previous study, we identified the *dilps* among several *Drosophila* IIS pathway genes that show increased expression in an IIS mutant, suggestive of such transcriptional feedback (Slack *et al.*, 2010).

Several dFOXO target genes have been well characterized in *Drosophila*, including the translational regulator *4E-BP*. We have demonstrated that *4E-BP* expression is upregulated in response to both reduced IIS and paraquat exposure in a dFOXO-dependent manner. Interestingly, *4E-BP* expression was also increased upon removal of dFOXO itself. It is possible that dFOXO functions to restrict basal *4E-BP* expression under normal conditions. Alternatively, the effects on *4E-BP* expression upon removal of dFOXO may be indirectly mediated via dFOXO-dependent effects on the activity of other transcription factors. *4E-BP* has recently been shown to be a potential target for the *Drosophila* FoxA transcription factor, forkhead (FKH), in response to low TOR signaling (Bulow *et al.*, 2010). *4E-BP* expression is suppressed by the loss of FKH activity and elevated upon FKH overexpression (Bulow *et al.*, 2010). dFOXO and FKH share the conserved forkhead DNA-binding domain, and so it is possible that they compete for binding at the same target genes. The increase in *4E-BP* expression observed in dFOXO mutants may therefore occur as a result of FKH-mediated transcriptional regulation. However, increased expression of other potential FKH target genes such as *cabut* and *CG6770* have not been observed in *dfoxo* mutants (N. Alic, C. Slack and L. Partridge, unpublished data).

Taken together, our data have shown that unlike in *C. elegans*, where all of the phenotypic effects of reduced IIS are dependent on DAF-16 activity, in *Drosophila*, several IIS-dependent phenotypes appear to be regulated, at least in part, through dFOXO-independent mechanisms. These results therefore have important implications when analyzing the requirements for IIS in particular phenotypic traits. In worms, the standard protocol would be to remove DAF-16 activity and look for abrogation of the response or phenotype. In flies, and possibly higher organisms, such experiments may prove misleading. For example, *dfoxo* mutants display a normal response to DR, but overexpression of the dominant negative insulin receptor or removal of dilps-2, -3 and -5 almost completely blocks the DR response (Grandison *et al.*, 2009; Broughton *et al.*, 2010; Gronke *et al.*, 2010). In conclusion, our data suggest that there is evolutionary divergence in the downstream effectors of IIS, and so in higher organisms, additional factors may act in concert with FOXOs to mediate the full response to reduced IIS.

## Experimental procedures

### Fly stocks and maintenance

The *dfoxo*^*Δ94*^ allele was generated by conventional imprecise excision using P[GT1]*foxo*^BG01018^ flies that carry an P[GT1] element transposon in the 5′upstream region of the *dfoxo* gene, approximately 130 nucleotides upstream of the *dfoxo* transcriptional start site (Dionne *et al.*, 2006). The 5′ and 3′ breakpoints of the *dfoxo*^*Δ94*^ deletion were mapped to the genomic sequence by PCR and sequencing. UASp-dFOXO transgenic flies for germline expression of dFOXO were generated using standard procedures. The P[GT1]*foxo*^BG01018^, *daughterless-GAL4* (*da-GAL4*), *UAS*-*InRDN* (*K1409A*), *UAS-Dp110DN* (*D954A*), *eyeless-GAL4* (*ey-GAL4*), and *mata*-*GAL4* stocks were obtained from the Bloomington Stock Centre. *daughterless-GeneSwitch* (*daGS*) was kindly provided by Veronique Monnier (Tricoire *et al.*, 2009). *InsP3-GAL4* was kindly provided by Michael Pankratz (Buch *et al.*, 2008). All stocks were backcrossed for at least 6 generations into the control *white*^*Dahomey*^ (*w*^*Dah*^*)* stock. *w*^*Dah*^ was derived by backcrossing *white*^*1118*^ into the outbred wild-type *Dahomey* background. Flies were raised and maintained on standard sugar/yeast medium (Bass *et al.*, 2007). Stocks were maintained, and experiments were conducted at 25 °C on a 12:12 hours light/dark cycle at constant humidity.

### Lifespan

Flies were reared at standard density (50 larvae per vial), allowed to mate for 24 h, sorted by sex, and then transferred to experimental vials at a density of ten flies per vial. Flies were transferred to fresh vials three times a week, and deaths were scored during transferral.

### Fecundity assays

For fecundity measurements, eggs were collected over a 24-h period and counted. Data are reported as the mean number of eggs laid per female ± SEM over this 24-h period.

### Paraquat and DDT assays

Flies were reared and housed as for lifespan experiments until 7 days of age, and then flies were starved for 5 h on 1% agar before being transferred to fly food containing either 20 mm paraquat or 0.03% (w/v) DDT.

### Growth analysis

Body weights of 7-day-old flies (*n* = 10 for each genotype) were measured using a precision balance. Wing areas were measured as previously described (Bohni *et al.*, 1999). For clonal analysis of growth, clones of *dfoxo*^*Δ94*^mutant cells were induced in the larval wing disks at 24–48 h after egg deposition by heat-shocking larvae of the genotype *y, w, hs-flp*/*w*; *FRT82*, *ubi-GFP*/*FRT82, dfoxo*^*Δ94*^ for 1 h at 37 °C. Larval wing disks were dissected out, fixed in 4% formaldehyde for 20 min at room temperature, and mounted in Vectashield mounting medium containing DAPI.

### Western blots

Western blots were carried out on protein extracts made from whole flies using a TCA-based extraction protocol. Equal amounts of protein as quantified using the Bio-Rad protein assay reagent were loaded onto SDS–PAGE gels and blotted according to standard protocols. Blots were probed with either anti-dFOXO antibody (Giannakou *et al.*, 2007) at a dilution of 1:5000 or anti-tubulin antibody (Sigma, Gillingham, UK) at 1:2500 dilution. Secondary antibodies conjugated to HRP (AbCam, Cambridge, UK) were used, and the signals were detected by chemiluminescence using the Enhanced ECL kit (GE, Amersham, UK).

### Quantitative RT-PCR

Total RNA was extracted from ten whole adult flies or 20 adult heads per genotype using standard Trizol (Invitrogen, Paisley, UK) protocols. cDNA was prepared using oligod(T) primer and Superscript II reverse transcriptase according to the manufacturer's protocol (Invitrogen). Quantitative RT-PCR was performed using the PRISM 7000 sequence detection system and Fast SYBR® Green PCR Master Mix (ABI, Warrington, UK). Relative quantities of transcripts were determined using the relative standard curve method and normalized to *actin5C*. Four or five independent RNA extractions were used for each genotype. Primer sequences are available upon request.

### Chromatin immunoprecipitation

Chromatin immunoprecipitations were carried out essentially as described (Slack *et al.*, 2010). For quantitative PCR, a suitable dilution of total chromatin and IP was used for the quantification using the PRISM 7000 sequence detection system and Fast SYBR® Green PCR Master Mix (ABI). For ChIP analysis, relative amounts of the target DNA recovered after ChIP compared with total chromatin were determined using two or three independent biological replicates. The relative proportion of DNA binding was calculated by dividing the proportion of DNA binding in the ChIP for a single region by the average recovered for all regions for that chromatin to normalize for plate–plate differences.

### Immunohistochemistry and confocal microscopy

Immunohistochemical analysis of dFOXO protein overexpression in whole mount ovaries of 7-day-old females was performed using the anti-dFOXO antibody at a dilution of 1:250 followed by Alexafluor 488 labeled anti-rabbit secondary antibody (Invitrogen). Nuclei were visualized using DAPI. Images were acquired using a Zeiss LSM 700 confocal microscope and zen (Zeiss, Welwyn Garden City, UK) software.

### Statistical analyses

Statistical analyses were performed using jmp (version 7) software (SAS Institute Inc., Cary, NC, USA). Lifespan data were subjected to survival analysis (Log-rank tests). Maximum lifespans were calculated as the median of the last surviving 10% of the population. Other data were tested for normality using the Shapiro–Wilk W test on studentized residuals (*Sokal & Rohlf, 1998*). One-way analyses of variance (anova) were performed, and planned comparisons of means were made using Tukey–Kramer HSD (Honestly Significant Difference) or Student's *t*-test.

## Supporting Information

**Fig. S1** (A) Survival curves of female *daGS*/+ flies +RU486 and −RU486. Experiments were started with 100 flies per condition. No significant difference in survival was detected between treatments by Log-rank test. (B) Average number of eggs laid per female after 7 days of RU486 treatment (+RU486) or untreated (−RU486). Data are presented as the mean number of eggs laid per female over a 24-h period ± SEM. Eggs were counted from ten separate vials and each vial contained ten females. No significant difference was detected between treatments by anova. (C) Survival of *daGS*/+flies in the presence of 20mM paraquat after 7 days of RU486 treatment (+RU486) or untreated (−RU486). Experiments were started with 100 flies per treatment. No significant difference was detected between treatments by Log-rank test. (D) Survival of *daGS*/+ flies in the presence of DDT after 7 days of RU486 treatment (+RU486) or untreated (−RU486). Experiments were started with 100 flies per treatment. No significant difference was detected between treatments by Log-rank test.

**Fig. S2** (A) Egg to adult development time in males and females of the indicated genotypes. Only the eclosion period of the adult flies is shown. Data are shown as percentage of flies eclosing. The delay in development observed in *dfoxo*^Δ94^ homozygotes is also observed in transheterozygotes of *dfoxo*^Δ94^/*dfoxo*^25^ and hemizygous *dfoxo*^Δ94^/*Df(3R)ED5624*. (Males: *wDahomey, n* = 114; *dfoxo*^Δ94^/+, *n* = 94; *dfoxo*^Δ94^/*dfoxo*^Δ94^, *n* = 106; *dfoxo*^25^/+, *n* = 84; *dfoxo*^Δ94^/*dfoxo*^25^, *n* = 112; *Df(3R)ED5624*/+, *n* = 98; *dfoxo*^Δ94^/*Df(3R)ED5624*, *n* = 84. Females: *wDahomey*, *n* = 107; *dfoxo*^Δ94^/+, *n* = 106; *dfoxo*^Δ94^/*dfoxo*^Δ94^, *n* = 116; *dfoxo*^25^/+, *n* = 114; *dfoxo*^Δ94^/*dfoxo*^25^, *n* = 118; *Df(3R)ED5624*/+, *n* = 93; *dfoxo*^Δ94^/*Df(3R)ED5624*, *n* = 99). (B) Body weights and wing areas as indicators of adult fly body size in females of the indicated genotypes. Data are presented as means ± SEM (*n* = 10 for each measurement). The reduced body size of *dfoxo*^Δ94^ homozygotes is also observed in transheterozygotes of *dfoxo*^25^/*dfoxo*^Δ94^ and hemizygous *Df(3R)ED5624*/*dfoxo*^Δ94^ (***P* < 0.05, *t*-test).

**Fig. S3** Effects of rapamycin treatment on the survivorship of *dfoxo*^Δ94^ mutants.

**Fig. S4** Lifespan and fecundity responses of *dfoxo*^Δ94^ mutant females to dietary restriction.

**Table S1** Median lifespans and statistical analysis of *dfoxo*^Δ94^ mutants and control flies across a food dilution series.

As a service to our authors and readers, this journal provides supporting information supplied by the authors. Such materials are peer-reviewed and may be re-organized for online delivery, but are not copy-edited or typeset. Technical support issues arising from supporting information (other than missing files) should be addressed to the authors.
