# Supplementary figures and images for: dFOXO-independent effects of reduced insulin-like signaling in Drosophila
Source: Aging Cell. 2011 Oct;10(5):735–48. doi: 10.1111/j.1474-9726.2011.00707.x (PMC3193374; doi:10.1111/j.1474-9726.2011.00707.x)

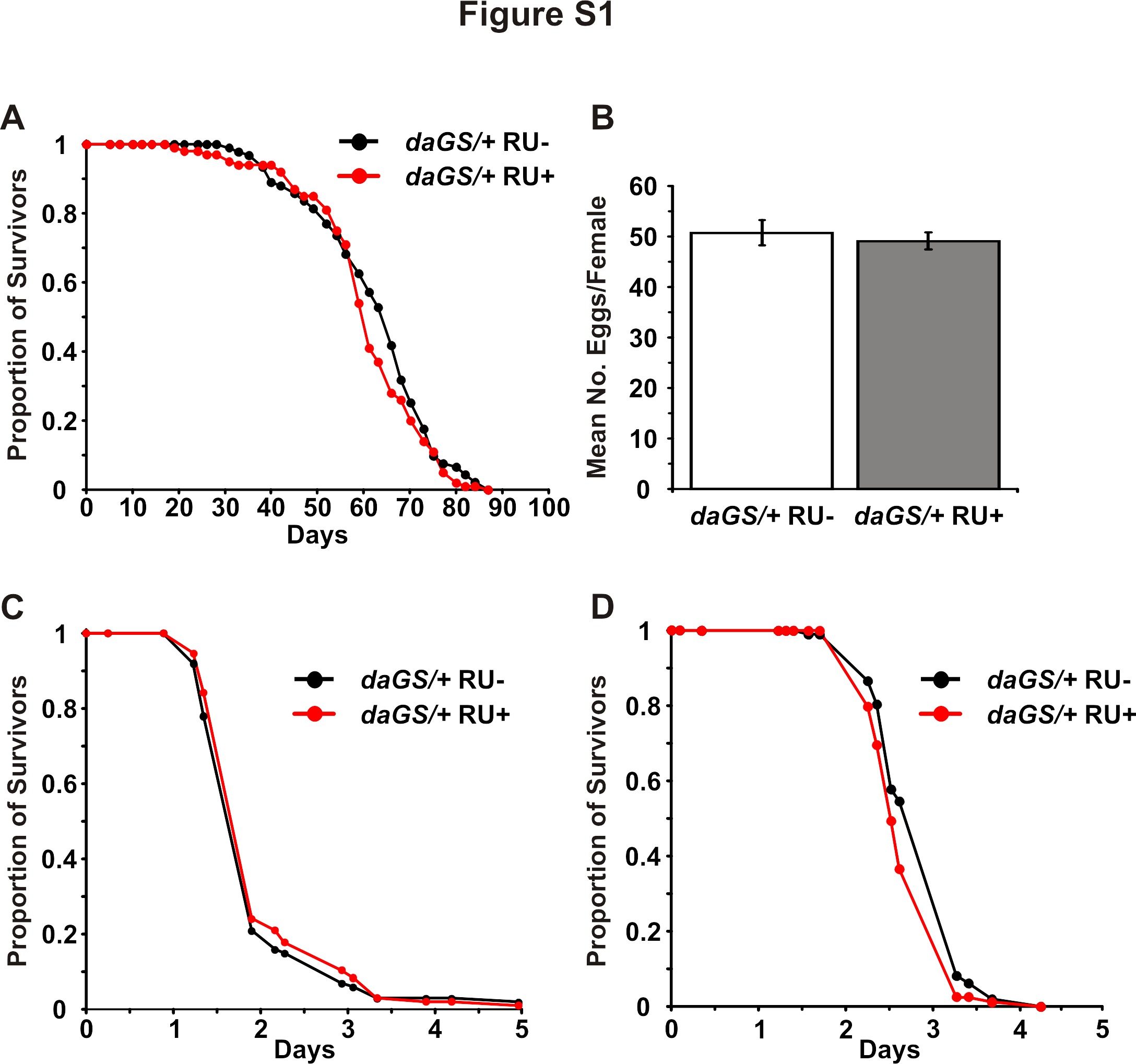

Supplement: Supplementary file 1 [file acel0010-0735-SD1.tif]

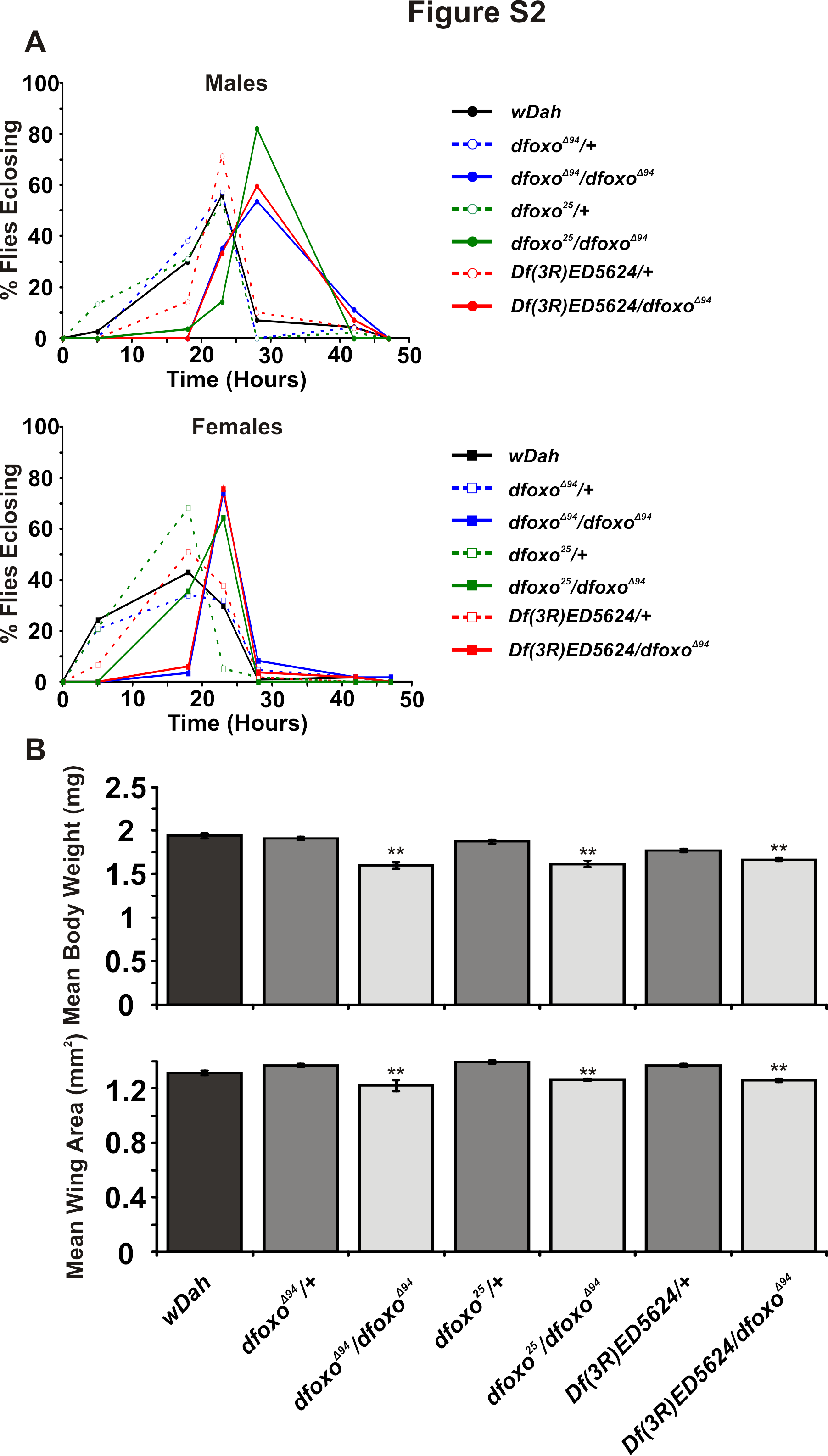

Supplement: Supplementary file 2 [file acel0010-0735-SD2.tif]

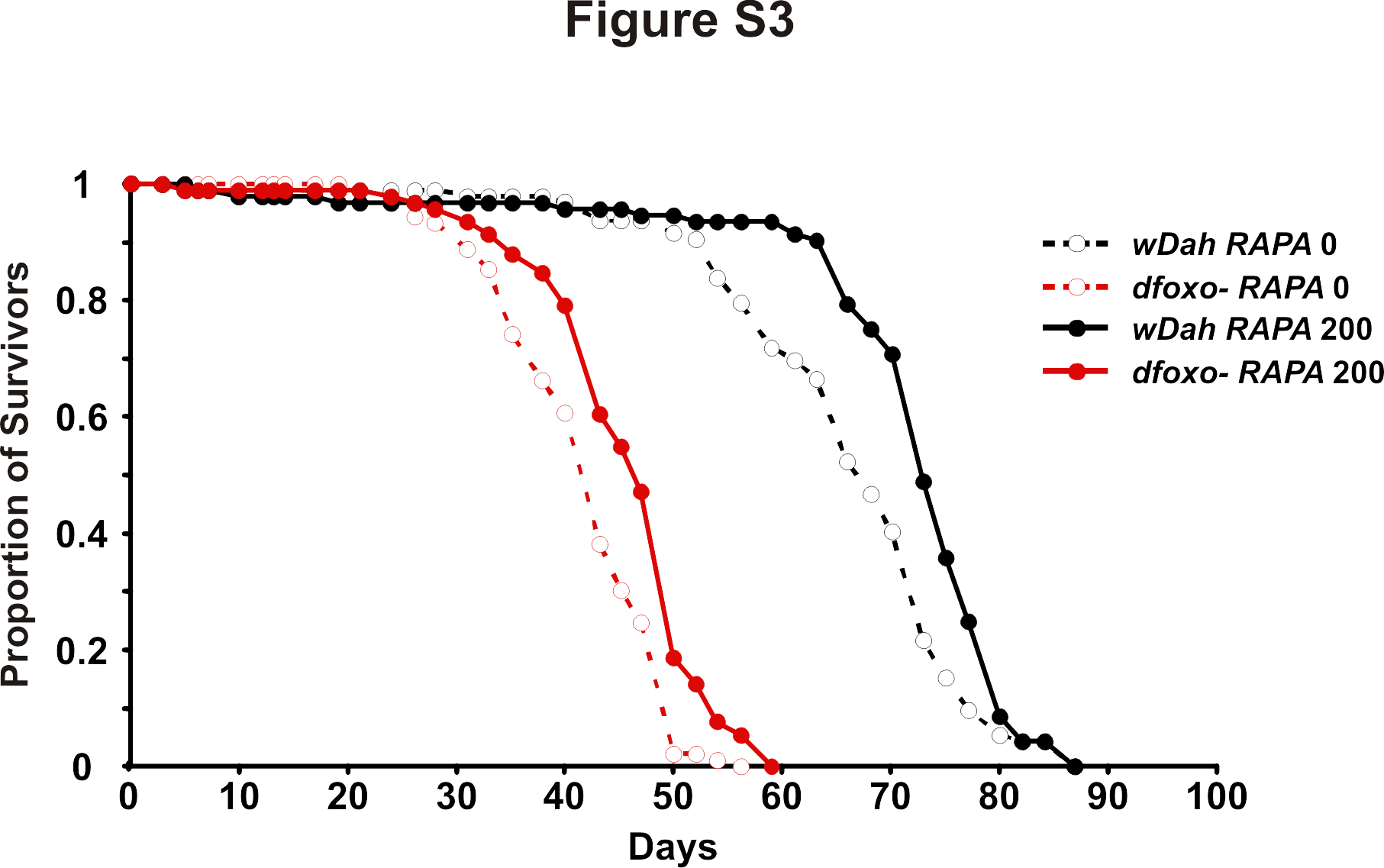

Supplement: Supplementary file 3 [file acel0010-0735-SD3.tif]

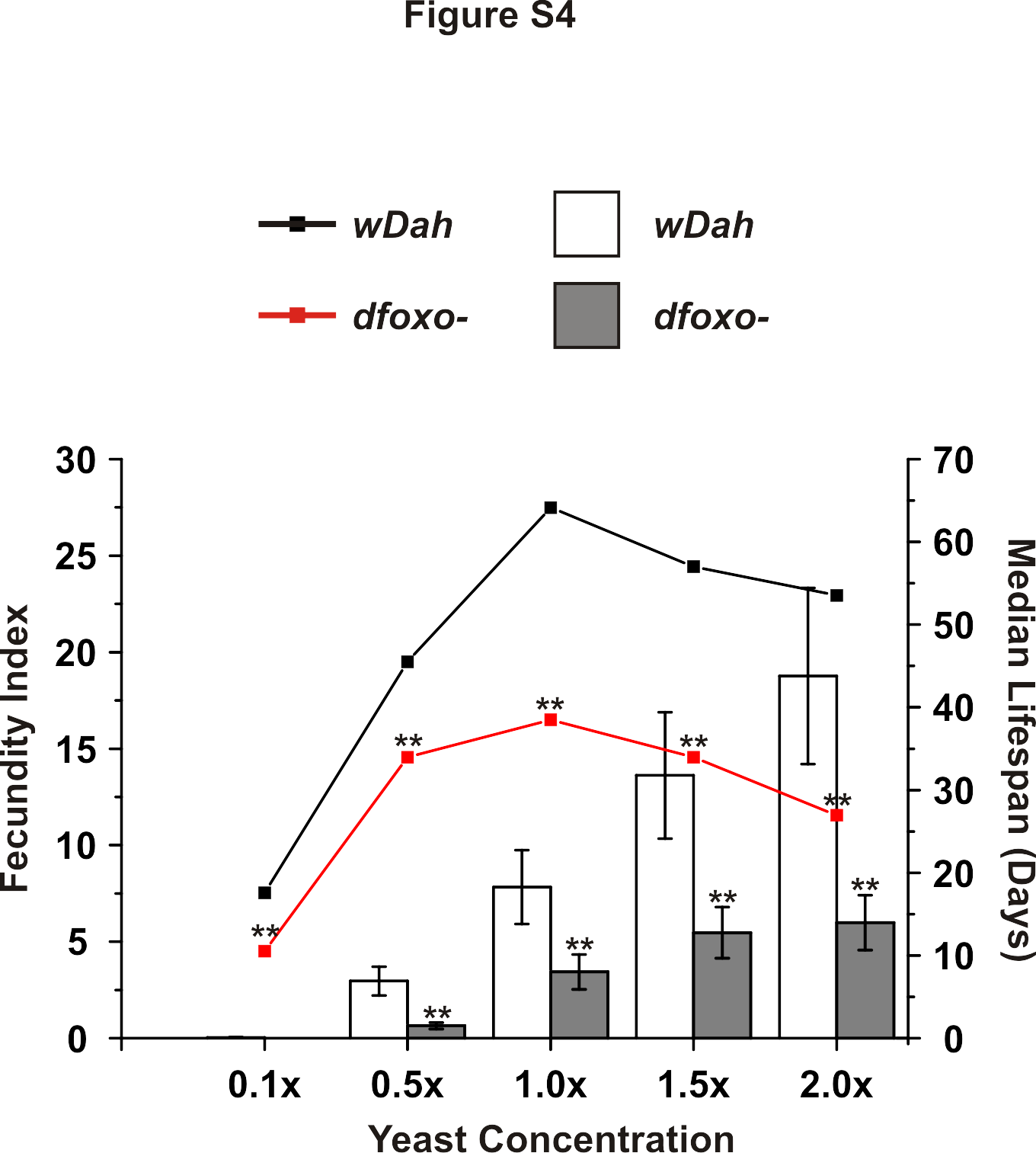

Supplement: Supplementary file 4 [file acel0010-0735-SD4.tif]
