# Supplementary material for: dFOXO-independent effects of reduced insulin-like signaling in Drosophila
Source: Aging Cell. 2011 Oct;10(5):735–48. doi: 10.1111/j.1474-9726.2011.00707.x (PMC3193374; doi:10.1111/j.1474-9726.2011.00707.x)
Supplement: Supplementary file 5 [file acel0010-0735-SD5.doc]

**Supplementary Table 1. Median lifespans and statistical analysis of *dfoxo^Δ94^* mutants and control flies across a food dilution series.**

| **Genotype** | **Yeast concentration** | | | | |
| --- | --- | --- | --- | --- | --- |
|  | **0.1x** | **0.5x** | **1.0x** | **1.5x** | **2.0x** |
| ***wDahomey*** | 17  (*n =* 96) | 45  (*n =* 94) | 64 †  (*n =* 87) | 57  (*n =* 89) | 53  (*n =* 92) |
| ***dfoxo^Δ94^*** | 10  (*n =* 98) | 34  (n =9 4) | 38 †  (*n =* 91) | 34  (*n =* 97) | 27  (*n =* 87) |
| ***p*-values** | <0.0001 | <0.0001 | <0.0001 | <0.0001 | <0.0001 |

*wDahomey* control and *dfoxo^Δ94^* homozygous mutant females were subjected to survival analysis across a yeast food dilution series. Table shows median lifespan; *n =* number of flies in group; *p-*values were calculated using Log-rank tests comparing the survival curves of *wDahomey* and *dfoxo^Δ94^* flies at each yeast concentration dilution; †Food dilution at which median lifespan peaked.
